# Characterization and gene expression analysis reveal universal stress proteins respond to abiotic stress in *Gossypium hirsutum*

**DOI:** 10.1186/s12864-023-09955-5

**Published:** 2024-01-23

**Authors:** Yunqing Li, Ao Zheng, Zhuang Li, Hu Wang, Jing Wang, Zhanghui Dong, Lina Yao, Xiao Han, Fei Wei

**Affiliations:** 1 College of Ecology, Lishui University, Lishui, 323000 China; 2 College of Plant Science and Technology, Huazhong Agricultural University, Wuhan, 430070 China; 3 College of Mathematics and Physics, Henan University of Urban Construction, Pingdingshan, 467000 China; 4 Shijiazhuang Academy of Agriculture and Forestry Sciences, Shijiazhuang, 050041 China; 5 Zhengzhou Research Base, National Key Laboratory of Cotton Bio-breeding and Integrated Utilization, Zhengzhou University, Zhengzhou, 450001 China

**Keywords:** Universal stress protein, *Gossypium hirsutum*, Abiotic stress

## Abstract

**Background:**

Universal stress proteins (USPs) are a class of stress-induced proteins that play a crucial role in biotic and abiotic stress responses. These proteins have previously been reported to participate directly in responses to various stress and protect plants against unfavorable environmental conditions. However, there is limited research on *USPs* in cotton, and systematic characterization of USPs in *Gossypium* species is lacking.

**Results:**

In the present study, the *USP* genes in *Gossypium hirsutum* were systematically identified and classified into six distinct subfamilies. The expansion of *USPs* in *Gossypium* species is mainly caused by dispersed duplication and whole genome duplication. Notably, the *USPs* that have expanded through allotetraploidization events are highly conserved in the allotetraploid species. The promoter regions of *GhUSPs* contain a diverse range of *cis*-acting elements associated with stress response. The RNA-Seq analysis and RT-qPCR assays revealed a significant induction of numerous *GhUSPs* expressions in response to various abiotic stresses. The co-expression network of *GhUSPs* revealed their involvement in stress response.

**Conclusions:**

This study systematically analyzed the biological characteristics of *GhUSPs* and their response to abiotic stress. These findings serve as a theoretical basis for facilitating the breeding of cotton varieties in future research.

**Supplementary Information:**

The online version contains supplementary material available at 10.1186/s12864-023-09955-5.

## Background

The initial discovery of the universal stress protein (USP) was documented in *Escherichia coli*. As the name suggests, USP proteins are known to have a significant impact on various biotic and abiotic stress factors [1, 2]. The subsequent studies have demonstrated the widespread presence of USP proteins in a variety of organisms, including bacteria, archaea, fungi, protists, plants, and animals [3, 4].

In the field of plant biology, USP proteins play a crucial role in responding to both abiotic and biotic stress. In *Arabidopsis*, a significant up-regulation of the expression levels of *AtUSP19* (At3g62550) and *AtUSP17* (At3g53990) wereobserved in drought treatment [5]. Subsequently, the over-expression of *AtUSP17* was found to enhance the resistance to extreme temperatures (low temperature and heat shock), oxidative stress, and pathogenic infection [6–8]. In *Solanum pennellii*, *SpUSP* was significantly upregulated in response to extreme temperatures, mechanical damage, and phytohormones (such as abscisic acid, gibberellic acid, and ethylene). Furthermore, it was found that overexpression of *SpUSP* could enhance drought tolerance and improve photosynthetic efficiency [9]. In *Solanum lycopersicum*, *SlRd2* could interact with *SlCipk6* and effectively improve the tolerance to both salt and osmotic stress [10]. In *Oryza sativa*, *OsUsp1* plays a critical role in the activation of ethylene signaling pathway in response to plant hypoxia [11]. In *Salicornia brachiata*, the ectopic expression of *SbUSP1* in *Nicotiana tabacum* significantly enhanced salt tolerance [12]. In *Morus alba*, overexpression of *MaUSP1* in *N. tabacum* enhanced tolerance to drought, salinity, and oxidative stress [12]. In the case of *Medicago falcata*, overexpression of *MfUSP1* enhanced various abiotic stresses, including freezing, salinity, and osmotic stress [13]. In addition, *USPs* participate in the growth and development of plants. Some *USP* genes were found to play a role in the seed germination process of *A. thaliana* [14]. Some *USPs* have been demonstrated to participate in the process of fruit ripening through the modulation of ethylene-mediated signaling pathways [5].

The *Gossypium* species is an ideal model to study polyploidization. Compared to the genome of *Theobroma cacao*, the *Gossypium* species underwent one more round of whole genome duplication (WGD) event approximately 60 million years ago (MYA) [15, 16]. Around 1 ~ 1.5 million MYA, *Gossypium hirsutum* (AD1) originated from the transoceanic hybridization between *Gossypium arboreum* (A2) and *Gossypium raimondii* (D5) [15, 17]. *G. hirsutum*, commonly known as upland cotton, dominates over 95% of global cotton cultivation due to its superior fiber and oil quality for worldwide industries. Nevertheless, in light of the escalating impacts of global warming and drastic weather changes, the cotton production is constrained by multiple abiotic stresses, such as drought, low temperature, and salinity. Thus, it is indispensable to mine the genes associated with abiotic tolerance. In previous research, the *USP* genes have been genome-wide identified in many plant species, and some of them have proven to play a vital role in the resistance of abiotic stress. However, the identification of the *USP* genes in cotton is still lacking [18–21].

In this study, the *USP* genes in four *Gossypium* species were systematically identified, and their evolutionary relationship and gene structure were analyzed. The *USP* genes in *Gossypium* species were mainly expanded by WGD and allotetraploidization events, and they were under purified selection during the evolution. The analysis of the *cis*-elements showed that *GhUSPs* were involved in abiotic stress. In addition, by performing the RNA-seq analysis and the qRT-PCR assays, we found that many *GhUSPs* were induced by low temperature stress. The WGCNA analysis showed that *GhUSPs* could co-express with many transcription factors in a salt and PEG stress-related network. This study provides a comprehensive view of *USP* genes in cotton, which will provide potential genes to evaluate the resistance to abiotic stresses.

## Results

### Genome-wide identification and characterization of *GhUSP* genes in cotton

**Fig. 1:**
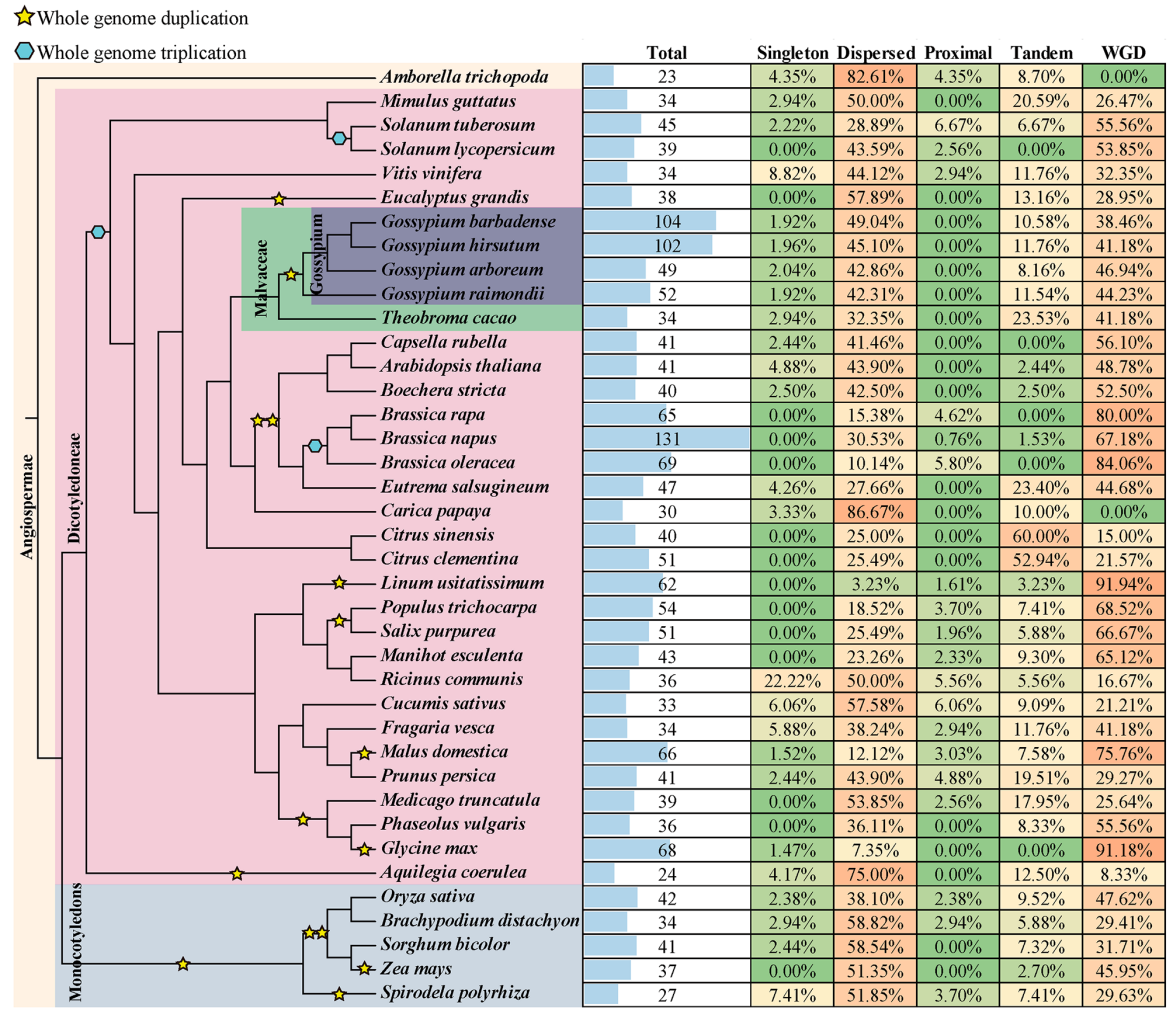
The identified *USP* genes in chosen plant genome and the related duplication events. The phylogenetic tree shown in the left side was obtained from the TimeTree website (http://www.timetree.org/), and the yellow pentagram and blue hexagon represented genome duplication event (WGD) and whole genome triplication event (WGT), respectively

The comprehensive analysis of genome-wide identification and conserved domain analysis revealed that the *USP* genes are prevalent in both monocotyledonous and dicotyledonous plant species. However, the number of *USP* genes, exhibited significant variation across different plant species, ranging from 23 to 131 (Fig. 1). A total of 49, 52, 102, and 104 *USP* genes were identified in *G. arboreum*(A2, 2n = 2x = 26), *G. raimondii* (D5, 2n = 2x = 26), *G. hirsutum* (AD1, 2n = 4x = 52), and *G. barbadense* (AD2, 2n = 4x = 52), the number of *USP* genes in the two tetraploid *Gossypium* species is nearly equal to the combined number of *USP* genes in the two diploid *Gossypium* species (Table S1). According to their genomic location, the USP members of *Gossypium* species were designated as *GaUSP1* to *GaUSP49*, *GrUSP1* to *GrUSP52*, *GhUSP1* to *GhUSP102*, and *GbUSP1* to *GbUSP104*, respectively. The protein length, protein molecular weight (MWs), isoelectric point (pIs), protein hydrophilicity and hydrophobicity of allotetraploid cotton and their diploid progenitors exhibited similar average distributions. (Table S1 and Fig. S1).

### Phylogenetic analysis and structure analysis of USP members

**Fig. 2:**
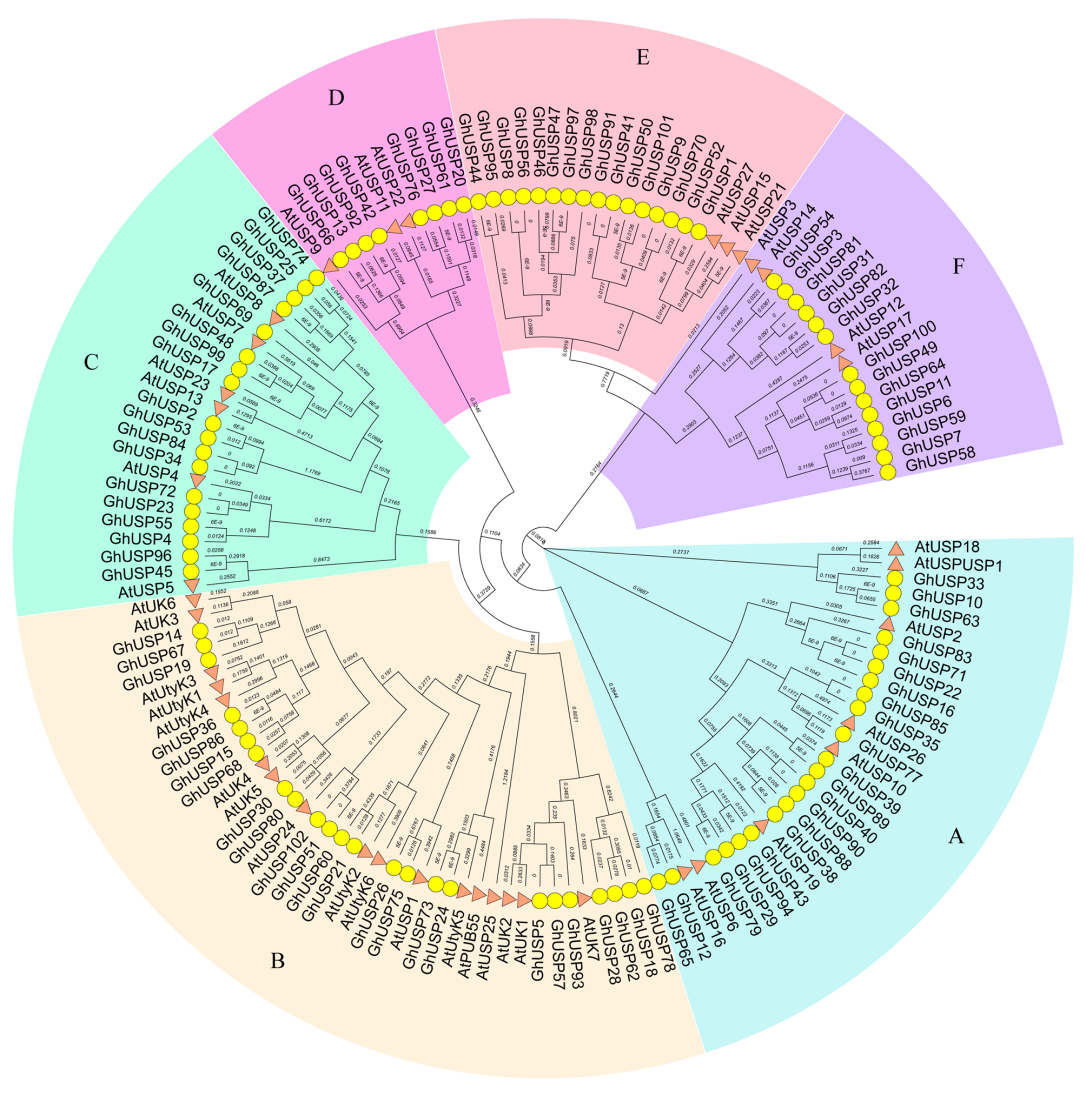
Phylogenetic relationship of USP proteins. From **A** to **F**, six subfamilies are represented by different backgroud colors. The sequences of USP proteins in *A.thaliana* and *G.hirsutum* were used to construct the phylogenetic tree were marked with pink triangle and yellow circle, respectively

As shown in the phylogenetic tree, the USP proteins of *G. hirsutum* can be classified into six distinct subfamilies, labeled as subfamilies A to F. Each subfamily contained multiple members from *G. hirsutum* and at least one member from the *AtUSPs*, which suggests that no subfamily specific to *Gossypium* was identified. Among the previously mentioned subfamilies, subfamily B had the largest number of members, accounting for 28.6% of the total. In contrast, subfamily D accounted for a significantly smaller portion, representing only 7.6% of the total (Fig. 2).

**Fig. 3:**
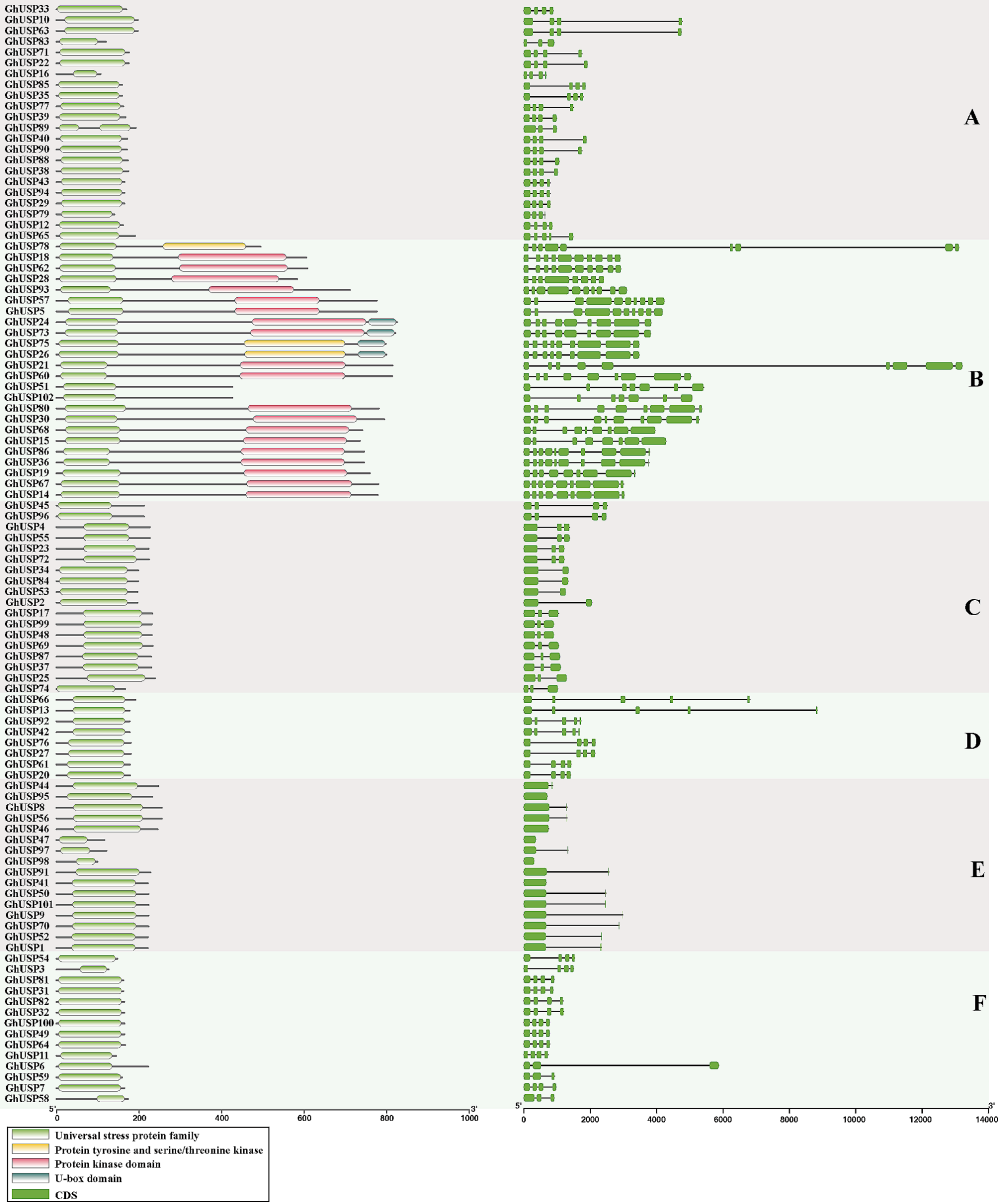
Conserved domain and gene structure analysis of USP members in *G.hirsutum*. The identified domains in USP proteins are represented by the boxes in different colors. The exons and introns of the *USP* genes are represented by green boxes and black lines, respectively. The scale at the bottom indicates the length of proteins and genes, respectively

The analysis of the protein domains revealed that all GhUSP proteins possessed a single USP domain, the majority of which span almost the entire length of the protein sequence. However, certain members of subfamily B were found to possess the protein tyrosine and serine/threonine kinase, protein kinase domain, and U-box domain, suggesting that they may serve a distinct purpose (Fig. 3). On the other hand, the gene structure (exon-intron structure) of the *GhUSPs* also varied distinctly in different subfamilies. All of the members within subfamily E contained 0 ~ 1 intron, while the members in subfamily B contained 6 ~ 9 introns (Fig. 3), suggesting that the gene structure and protein architecture of *GhUSPs* are conserved within each specific subfamily, which is consistent with the classification of the phylogenetic analysis (Figs. 2 and 3).

### Chromosome location, gene Dduplication, and selection pressure of *USP* genes

**Fig. 4:**
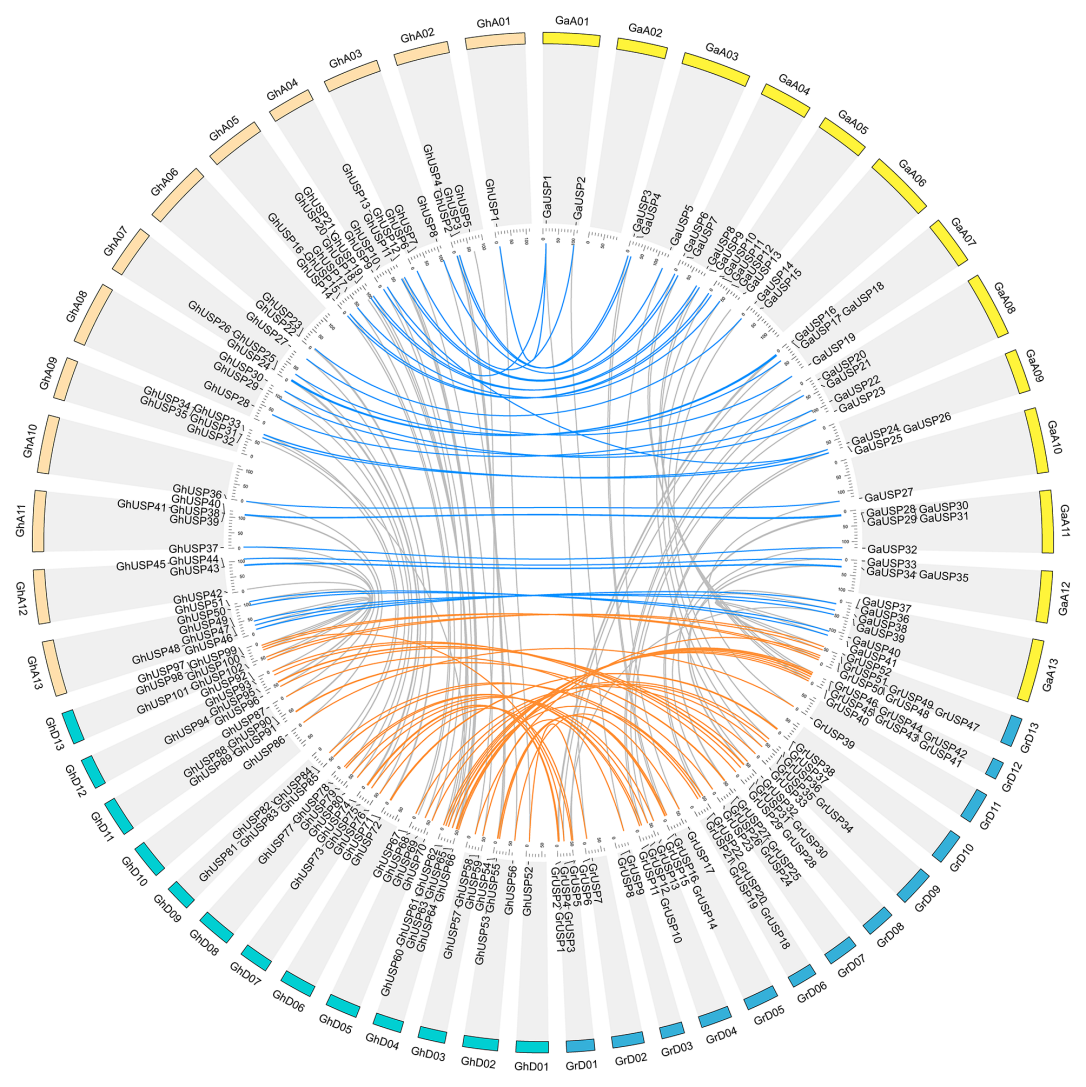
Chromosome distribution and collinearity analysis of *USP* genes in *G.hirsutum*, *G.arboreum*, and *G.raimondii*. Collinear gene pairs between *GhUSP* genes and *GaUSP* genes, between *GhUSP* and *GrUSP* genes, are represented by blue and orange lines, respectively

The *USP* genes of four *Gossypium* species are unevenly distributed in all chromosomes (Fig. 4 and Table S1). The *USP* genes in the genome of *G.arboreum* were predominantly found on chromosomes 13 and 11. The *USP* genes from *G. raimondii* were mainly distributed on chromosomes 13 and 12. For the allotetraploid species *G.hirsutum*, *USPs* were mainly detected in the A05, A13, D13, and D04. The chromosome location of the majority of the *GhUSPs* was conserved after the allopolyploidization events (Fig. 4).

To investigate the expansion pattern of *USP* genes, we further examined the duplication pattern. Multiple types of duplication events were detected in cotton’s *USP* genes, the dispersed duplication event and WGD event were found to be primary drivers of the expansion of *USP* genes *Gossypium* species (Fig. 1). Among the other selected plant species, the *USP* genes were mainly expanded through dispersed duplication (86.67% in *Carica papaya* and 75.00% in *Aquilegia coerulea*) and WGD event (91.94% in *Linum usitatissimum* and 91.18% in *Glycine max*) (Fig. 1). However, tandem duplication events played a significant role in the expansion of *USP* genes in certain cases, accounting for 60.0% and 52.94% of *USP* genes in *Citrus sinensis* and *Citrus clementina*, respectively (Fig. 1). These findings suggest that the expansion of *USP* genes in the majority of species was likely driven by dispersed events and WGD, whereas tandem duplication events may also contribute to the expansion of *USP* genes. Additionally, the allopolyploidization event was identified as a significant factor contributing to the expansion of *USP* genes in tetraploid cotton. The number of *USP* genes present in each tetraploid cotton is nearly equivalent to the sum of two diploid *Gossypium* species (Figs. 1 and 4). The similar result was also shown in the *Brassica* species. The number of *USP* genes in tetraploid species *B.napus* is roughly equal to the sum of diploid species *B.rapa* and *B.oleracea* (Fig. 1).

All the Ka/Ks ratios of gene pairs of *USP* genes in *Gossypium* species, resulting from various duplication events, are lower than 1. This observation suggests that the *USP* genes have likely undergone strong purifying selection pressure throughout their evolutionary history. Furthermore, it indicates that the protein functions of these genes may be conserved after the expansion (Table S2).

### *cis*-elements analysis and expression pattern of *GhUSPs*

Multiple *cis*-elements associated with stress response were detected in 2000 bp upstream of the start codon of *GhUSPs*, including STRE, MYB, and w-box (Fig. S2). Therefore, we speculated that *GhUSPs* may have a vital role in regulating the defense against the stress from the external environment. On the other hand, based on the analysis of the RNA-seq data, it was observed that the expression levels of numerous *GhUSPs* exhibited up-regulation under various abiotic stress conditions. Under cold stress conditions, the expression of several *GhUSPs* was upregulated, including *GhUSP11*, *29*, *32*, *43*, *64*, *82*, *88*, and *94* (Fig. S3A). After the salt treatment, the expression of many *GhUSPs* was highly induced, such as *GhUSP11*, *29*, *32*, *43*, *82*, *88*, and *94* (Fig. S3B). After the treatment with polyethylene glycol (PEG), the expression level of numerous *GhUSPs* was upregulated, including *GhUSP11*, *29*, *32*, *43*, *82*, *88*, and *94* (Fig. S3C).

**Fig. 5:**
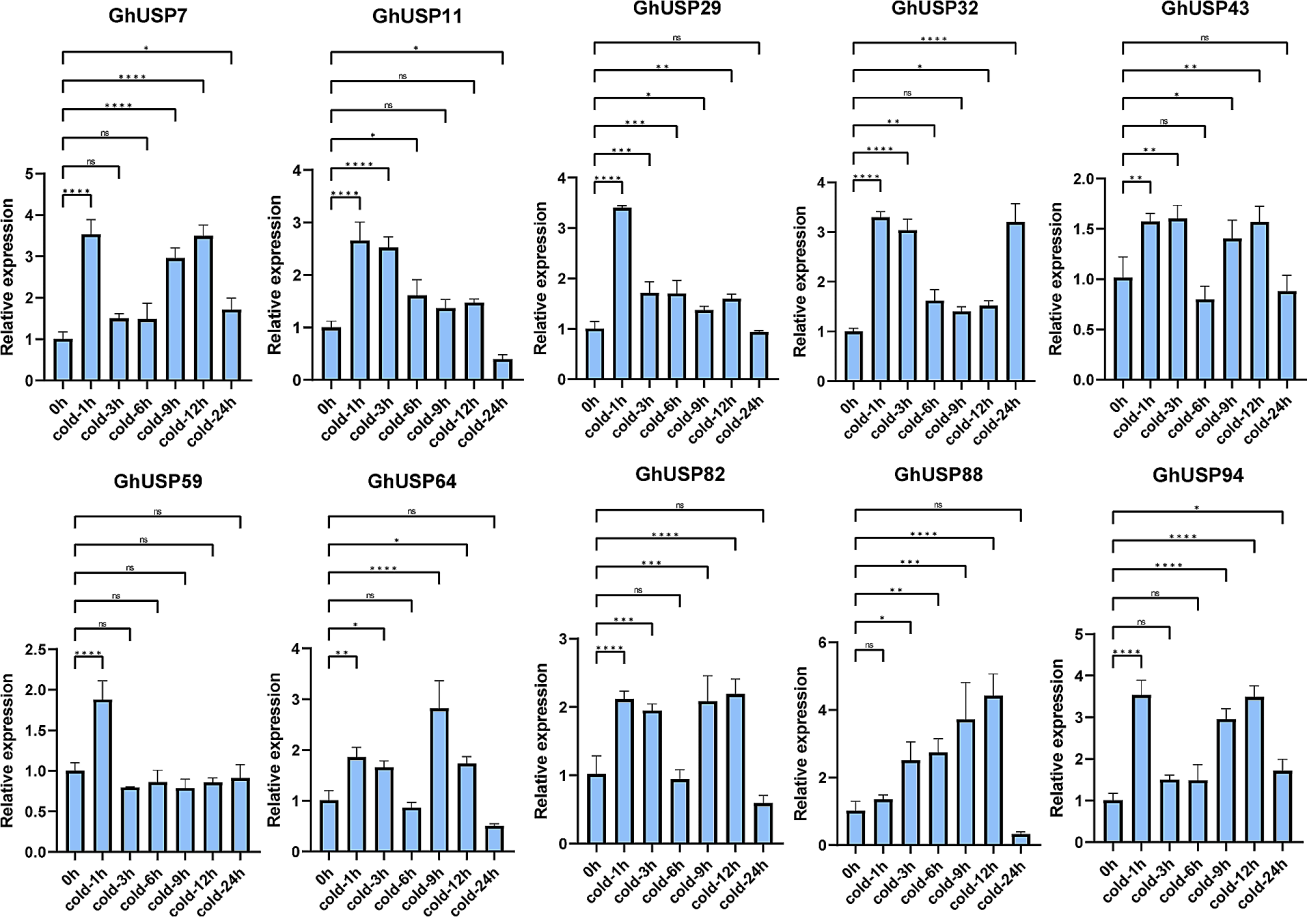
Expression analysis of *GhUSPs* in leaves under cold treatment via RT–qPCR. The samples were collected at 0 h, 1 h, 3 h, 6 h, 9 h, 12 and 24 h after cold treatment on TM-1 cotton seedlings. *GhActin7* was used as an internal reference. Error bars represent the standard deviation of three independent biological replicates. Statistically significant difference was evaluated by one-way ANOVA analysis (*P < 0.05, **P < 0.01, ***P < 0.001, and ****P < 0.0001). The primers used are listed in Table S3

**Fig. 6:**
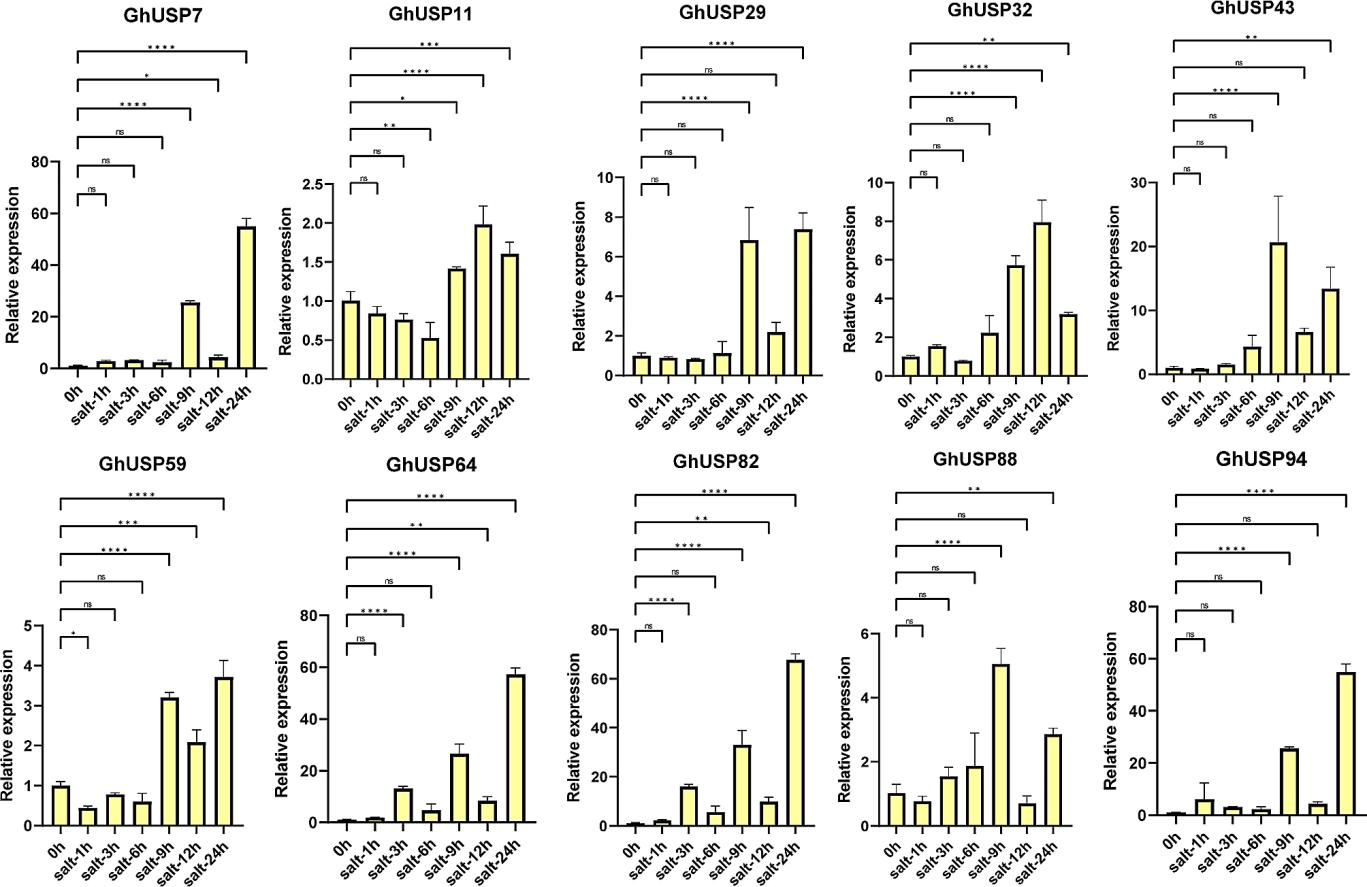
Expression analysis of *GhUSPs* in leaves under salt treatment via RT–qPCR. The samples were collected at 0 h, 1 h, 3 h, 6 h, 9 h, 12 and 24 h after salt treatment on TM-1 cotton seedlings. *GhActin7* was used as an internal reference. Error bars represent the standard deviation of three independent biological replicates. Statistically significant difference was evaluated by one-way ANOVA analysis (*P < 0.05, **P < 0.01, ***P < 0.001, and ****P < 0.0001). The primers used are listed in Table S3

**Fig. 7:**
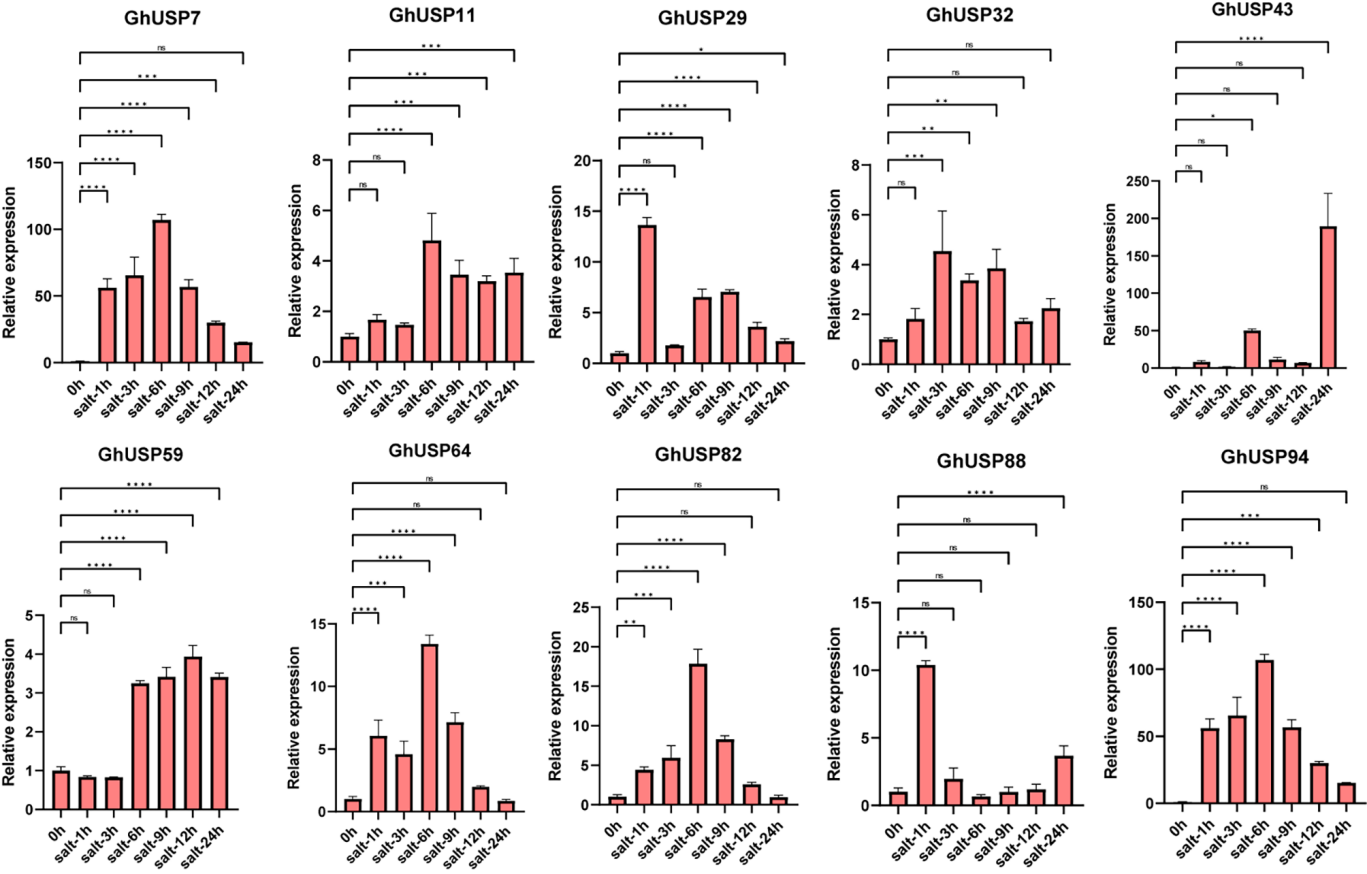
Expression analysis of *GhUSPs* in leaves under PEG treatment via RT–qPCR. The samples were collected at 0 h, 1 h, 3 h, 6 h, 9 h, 12 and 24 h after PEG treatment on TM-1 cotton seedlings. *GhActin7* was used as an internal reference. Error bars represent the standard deviation of three independent biological replicates. Statistically significant difference was evaluated by one-way ANOVA analysis (*P < 0.05, **P < 0.01, ***P < 0.001, and ****P < 0.0001). The primers used are listed in Table S3

To further confirm the expression pattern of *GhUSPs* under abiotic stress, the aforementioned *GhUSPs* were chosen to perform the RT-qPCR assays. Under the cold stress, *GhUSP11*, *29*, and *59* were up-regulated at the beginning. The expression of *GhUSP7* was induced at 1, 9, and 12 h while decreased to the normal expression at 3, 6, and 24 h, the expression of *GhUSP88* increasing continuously but decreased at 24 h (Fig. 5). For the salt treatment, the expression of most *GhUSPs* was highly induced, *GhUSP43* exhibited multiple times more relative expression than that in the control group (Fig. 6). For PEG stress, the expression of *GhUSP7*, *64*, *82*, and *94* continuously increased and peaked at 6 or 9 h but decreased at the following time points, the expression of the *GhUSP43* peaked at 24 h (Fig. 7). Taken together, these results suggest that *GhUSPs* might play potential regulatory roles in the response to abiotic stress.

### WGCNA network analysis of *GhUSPs* related to abiotic stress

Further WGCNA analysis showed that four and five modules were identified in PEG and salt stress, respectively. Among these molules, *GhUSPs* could act as hub genes within the co -expression. In the co-expression network related to salt stress, *GhUSP43* exhibits co-expression with numerous stress-related genes, including *GhZTP29* (GH_A08G0168), *GhNST1* (GH_D12G1658), and several members of the CYP450 gene family (GH_D09G0584, GH_D11G1438, GH_D11G1957, and GH_D12G0969) (Fig. S4A). In the co-expression network related to PEG stress, *GhUSP11*, *32*, *43*, and *94* were identified, and numerous transcription factors were also found in this network. It was shown that many genes associated with reactive oxygen species (ROS) signaling are also present in this network, such as *GhEGY3* (GH_D01G0096) and *GhRIPK* (GH_D13G2178) (Fig. S4B). Therefore, we speculated that *GhUSPs*, such as *GhUSP43*, might play a significant role in responding to salt and drought stress.

## Discussion

Cotton is often referred to as “white gold” and is one of the most economically significant crops globally, accounting for approximately 35% of the world’s annual demand for textile fiber [15]. Abiotic stresses, such as extreme temperatures, drought, and salinity, commonly pose a threat to the yield of cotton [22, 23]. Previous studies indicate that ubiquitin-specific proteases (USPs) play a significant role in responding to both abiotic and biotic stress responses [8–10, 13, 24, 25]. However, this gene family has not been systematically characterized in cotton.

In the present study, a comprehensive identification and analysis of *USP* genes was conducted. The *USP* genes were systematically identified in *G. arboretum*, *G. raimondii*, *G. barbadense*, and *G. hirsutum* (Fig. 1). The identified members were classified into six distinct subfamilies based on the results of phylogenetic analysis (Fig. 2). It is noteworthy that the conservation of the number of exons is observed within each subfamily, which is consistent with the classification of the *GhUSPs* (Figs. 2 and 3).

To effectively respond to dynamic environmental conditions, various types of gene duplication events have the potential to generate novel genes and functions [26, 27]. The *Gossypium* species is commonly used as a model system to study plant polyploidization, including allotetraploidization and WGD events [15, 16, 28]. The number of USP members in each allotetraploid species is nearly equivalent to thesum of the diploid parental species. Furthermore, the analysis of chromosome location revealed a consistent pattern in the diploid species (Fig. 4). Additionally, the WGD play a significant role in expanding the *USP* genes in *Gossypium* species, followed by dispersed duplication, and they have undergone purifying selection pressure (Fig. 1, Table S2). The *Brassica* species have also been served as excellent models for studying WGD and allotetraploidization events [29]. Similarly, the allotetraploid species *B. napus* exhibits a nearly cumulative count of *USP* genes when compared to the diploid species *B.rapa* and *B.olerecea* (Fig. 1). Additionally, the physical and chemical characteristics of the allotetraploid cotton were found to be similar to those of the diploid species (Table S1). These findings demonstrate the substantial contribution of polyploidization events, including WGD and allotetraploidization events to the expansion of the expansion of the *USP* genes in *Gossypium* species. These findings provide additional evidence to reinforce the previous assertion that allotetraploid cotton species have their origins in the natural hybridization of two diploid progenitors.

*cis*-regulatory elements have the potential to regulate gene expression during plant development and in response to environmental stimuli [30]. In the present study, an enrichment of MYB and MYC motifs was observed in the promoter sequences of *GhUSPs* (Fig. S2). Previous research has shown that MYB and MYC motifs in plants can respond to abiotic stress [31–33]. Furthermore, by analyzing the previous transcriptome data and subsequently confirming it through qRT-PCR assays, it was observed that numerous *GhUSPs* could respond to the cold, salt, and PEG stress. For instance, the expression of *GhUSP43* was upregulated in response to both salt and PEG treatments (Figs. 5, 6 and 7 and S3). Therefore, we propose a preliminary hypothesis that *GhUSPs* may potentially contribute to the modulating various abiotic stress factors.

WGCNA has been demonstrated as a cost-effective approach for identifying critical players within specific modules, thereby enabling the prediction of the function of novel genes [34]. In this study, an analysis was conducted on the transcriptome data obtained from a previous research. The analysis revealed that several *GhUSPs* were identified in the co-expression networks associated with salt and PEG stress [17] (Fig. S4, Table S4, and Table S5). Many genes identified within these networks have been found to be involved in responding to abiotic stress and the signaling process of ROS [35–40].

## Conclusions

In this study, we systematically analyzed the *GhUSP* gene family in cotton. A total of 102 *GhUSP* genes were identified. Their chromosome localization, evolutionary tree, linear analysis, gene duplication, selective pressure, gene structure, motif distribution, *cis*-acting elements, and WGCNA were analyzed and characterized. We further investigated the expression patterns of *GhUSP* genes in response to salt, drought, and cold treatment. Our findings provide valuable information and a theoretical framework for further research into their specific roles in various development processes and abiotic stress. This is of great significance for breeding cotton with resistance.

## Materials and methods

### The data retrieval and identification of USP proteins

In this study, we obtained the sequenced genomes of multiple plant species. Detailed information on these genomes is provided in Table S6. The Hidden Markov Model of the USP domain (PF00582) was obtained from the InterPro database (https://www.ebi.ac.uk/interpro/), and the USP proteins were scanned by the Hmmsearch program [41, 42]. Next, the Interproscan software was used to identify the domain of the above protein sequences, the sequences without the USP domain were removed [42]. The protein properties of USP proteins were predicted by the ProtParam module in Biopython [43].

### Sequence alignment and phylogenetic analysis

The identified USP proteins from *A.thaliana* and *G.hirsutum* were analyzed for multiple sequence alignment using MAFFT software [44]. Next, by employing trimAl software, the gaps in the alignment results were further removed [45]. Based on the above results, the phylogenetic tree was generated by FastTree software [46]. Finally, the phylogenetic tree was visualized by the EvolView v3.0 [47].

### The analysis of chromosome location and gene collinearity

By extracting the genomic annotation data (gff/gtf files) of the USP members, the chromosomal locations of USP members from four *Gossypium* species were retrieved. By performing the BLASTP and MCScanX program, the collinearity of gene pairs within the USP members was identified, and the above results were visualized by circos software [48–50].

The duplication event related to USP members was identified by MCScanX [51]. By performing the Dupgen_finder pipeline, the gene pairs generated by the above duplication events were retrieved [52]. By employing the ParaAT pipeline, the protein and coding sequences of USP members were aligned to generate the alignment results with the AXT format [53]. The synonymous rate (Ks) and nonsynonymous rate (Ka) values were analyzed by Kaks_calculator 2.0 [54].

### *cis*-acting element analysis

Based on the annotation file (gtf file), the 2000 bp from the upstream genomic DNA sequences of *GhUSP* genes were obtained. The cis-acting elements were identified by the PlantCARE website (http://bioinformatics.psb.ugent.be/webtools/plantcare/html/) [55].

### RNA-seq and WGCNA analysis

The transcriptome data (accession number: PRJNA490626) was retrieved from SRA database, including four-week-old seedlings under the salt, PEG, 4°C, and 37°C treatments for 0, 3, 6, 9, 12, and 24 h [17, 56]. By performing the trimmomatic (v0.3.9) software, the low-quality reads were filtered [57]. The ZJU2.1 version of *G.hirsutum* genome was chosen as the reference genome in this study, and the index file was built by HISAT2 (version 2.1.0) software [58]. The clean reads were mapped to the reference genome by performing the HISAT software [17, 58]. The transcripts were assembled by Stringtie (v2.0) software [59]. The FPKM (Fragments Per Kilobase of transcript per Million mapped reads) value was generated by the R software.

The differentially expressed genes (DEGs) were analyzed by edgeR package in R software, which is based on the rule of| logFC| > 1 and *p*-value < 0.05 [60]. The modules dividing and weighted gene co-expression network constructing were performed by the WGCNA (version 1.69) package, the weight value was calculated by the pickSoftThreshold program [61]. The networks were visualized by the CytoHubba package of Cytoscape software (v3.9.1) [62].

### Plant materials and treatments

The seeds of *G. hirsutum* cultivar TM-1 were grown in the growth room with a condition of 16 h light at 27 °C/8 h dark at 22 °C. After three weeks, the seedlings at the three-leaf stage were treated with cold (4 °C), salt (200 mM NaCl), and simulate drought stress (30% PEG6000), the leaves were collected at 0 h (control), 1 h, 3 h, 6 h, 9 h, 12 h, and 24 h. All samples were immediately frozen in liquid nitrogen and stored in a − 80 °C refrigerator for subsequent RNA extraction.

### The RNA extraction and RT–qPCR analysis

The RNA of the collected samples was extracted using the Vazyme RNA extraction kit (FastPure Plant Total RNA Isolation Kit, RC411-C1). The RNA reverse transcription used the Vazyme reverse transcription kit (HiScript II Q RT SuperMix for qPCR, R223-01). Next, the RT–qPCR assays were performed on a Thermofisher ABI instrument (7500 Real Time PCR System). The reaction procedure is: preheat denaturation of 95 °C for 10 min; 40 cycles of 95 °C for 5 s, 60 °C for 15 s; 95 °C for 15 s, 60 °C for 1 min for the melting curve programs. The reaction system is: 10.0 µL 2xUltra SYBR Mixture, 0.6 µL (10 µmol L^− 1^) PCR forward primer (10 µM), 0.6 µL (10 µmol L^− 1^) PCR reverse primer (10 µM), 0.8 µL cDNA templates and 8 µL sterile ddH2O. *G.hirsutum* Actin (*GhActin*) was used as the internal reference gene, and the relative expression levels was calculated by the 2^−△△CT^ algorithm [63]. The primers are listed in Table S3.

### Statistical analysis

Data are expressed as means ± SD. Statistical tests were using GraphPad Prism 8 software performed one-way ANOVA method (*P < 0.05, **P < 0.01, ***P < 0.001, and ****P < 0.0001).

### Electronic Supplementary Material

Below is the link to the electronic supplementary material.

**Supplementary Material 1**: Table S1. Basic information on *USP* genes in *Gossypium* species

**Supplementary Material 2**: Table S2. Ka/Ks ratios for duplicated *GhUSP* gene pairs

**Supplementary Material 3**: Table S3. Primers used in RT–qPCR

**Supplementary Material 4**: Table S4. The information of genes displayed in the WGNCA network related to salt stress

**Supplementary Material 5**: Table S5. The information of genes displayed in the WGNCA network related to PEG stress

**Supplementary Material 6**: Table S6. The information of the chosen plant genome used in this study

**Supplementary Material 7**: Fig.S1. The physical and chemical parameters *USP* proteins in *Gossypium* species. The original data was list in Table S1

**Supplementary Material 8**: Fig.S2. Analysis of *cis*-elements in promoters of *GhUSP* genes. The color scale represents the number of the identified *cis*-elements retrieved from the PLANTCARE website (http://bioinformatics.psb.ugent.be/webtools/plantcare/html/). Red indicates a high number level and blue indicates a low number level

**Supplementary Material 9**: Fig.S3. The transcript profiling of *GhUSP* genes under the (A) cold, (B) salt, and (C) PEG treatment. The scale bar means the scaled expression level, red indicates a high expression level and white indicates a low expression level. The number displayed in each boxes represent log2 transformed FPKM values. The raw RNA-seq data was retrieved from a previous study (accession number: PRJCA004262)

**Supplementary Material 10**: Fig.S4. Gene co-expression network contained *GhUSPs* related to abiotic stress. (A) The co-expression network under salt treatment. (B) The co-expression network under PEG treatment. The red triangle symbolizes *GhUSPs*. The displayed genes and their corresponding description are presented in Table S4 and Table S5

## Data Availability

The RNA-seq data used in this study were retrieved from the NCBI Sequence Read Archive (SRA) database (https://www.ncbi.nlm.nih.gov/sra) under the accession code PRJNA490626.
