# Supplementary figures and images for: Characterization and gene expression analysis reveal universal stress proteins respond to abiotic stress in Gossypium hirsutum
Source: BMC Genomics. 2024 Jan 23;25:98. doi: 10.1186/s12864-023-09955-5 (PMC10804864; doi:10.1186/s12864-023-09955-5)

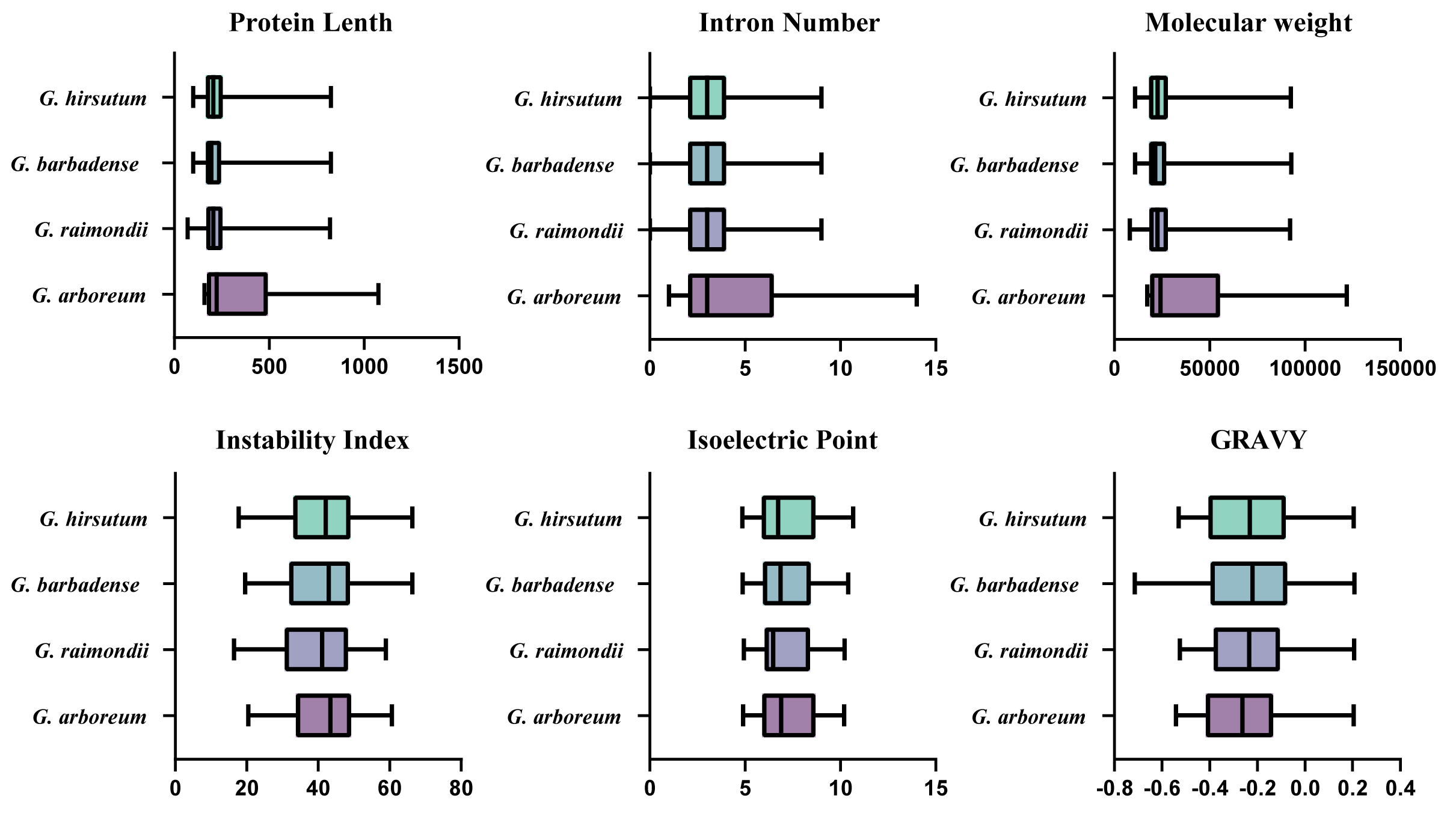

Supplement: Supplementary file 7 — Supplementary Material 7: Fig.S1. The physical and chemical parameters USP proteins in Gossypium species. The original data was list in Table S1 [file 12864_2023_9955_MOESM7_ESM.png]

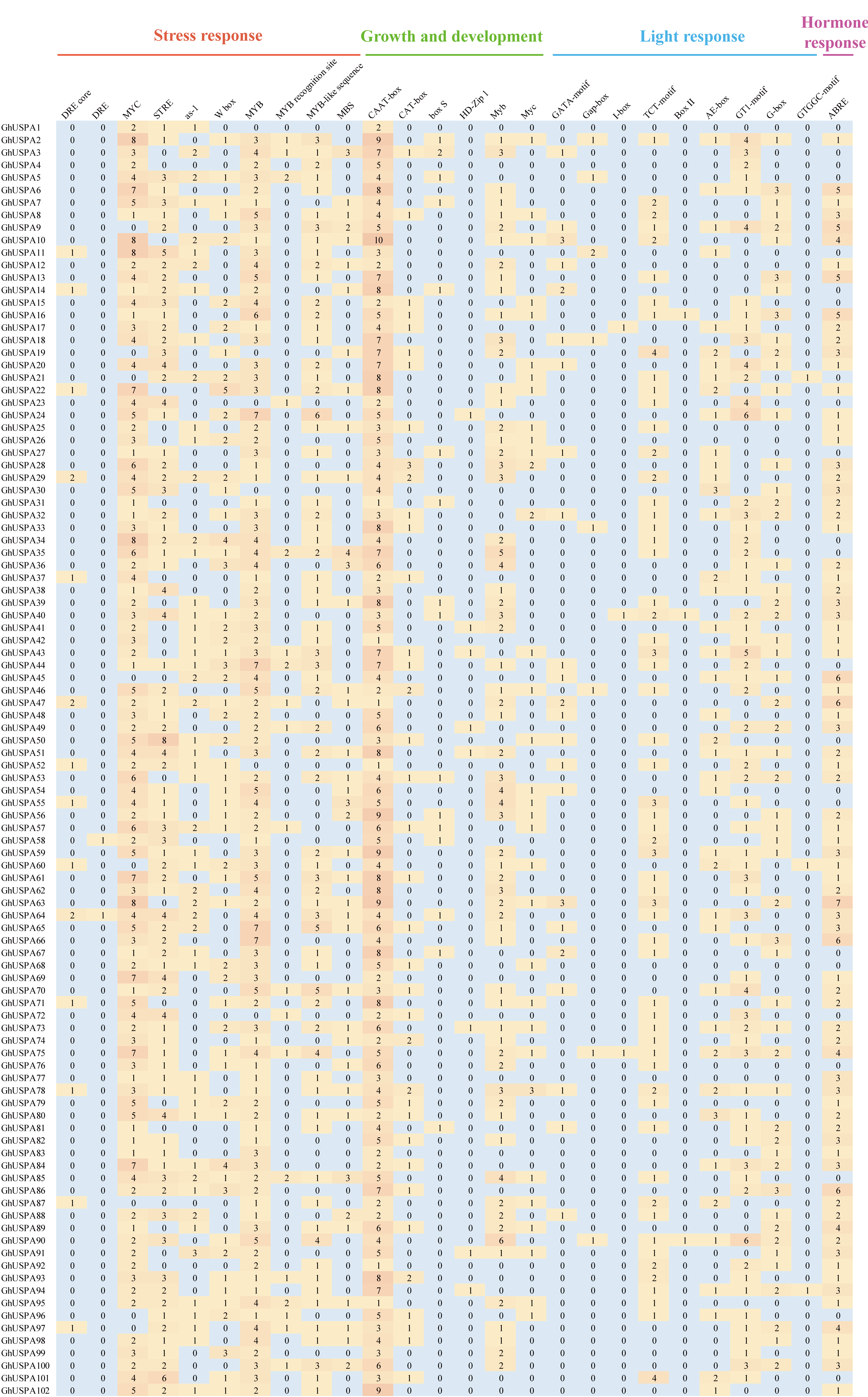

Supplement: Supplementary file 8 — Supplementary Material 8: Fig.S2. Analysis of cis-elements in promoters of GhUSP genes. The color scale represents the number of the identified cis-elements retrieved from the PLANTCARE website (http://bioinformatics.psb.ugent.be/webtools/plantcare/html/). Red indicates a high number level and blue indicates a low number level [file 12864_2023_9955_MOESM8_ESM.png]

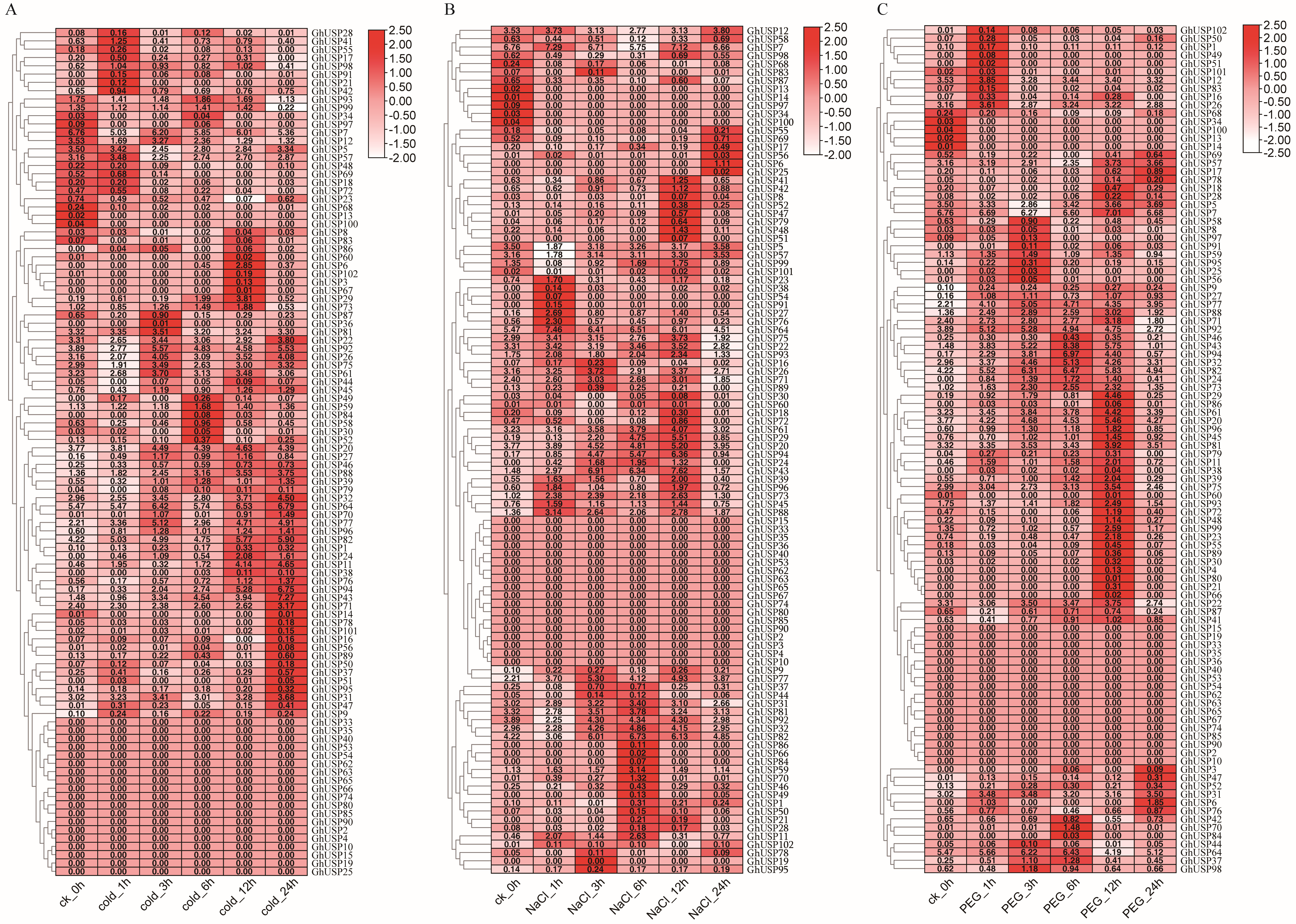

Supplement: Supplementary file 9 — Supplementary Material 9: Fig.S3. The transcript profiling of GhUSP genes under the (A) cold, (B) salt, and (C) PEG treatment. The scale bar means the scaled expression level, red indicates a high expression level and white indicates a low expression level. The number displayed in each boxes represent log2 transformed FPKM values. The raw RNA-seq data was retrieved from a previous study (accession number: PRJCA004262) [file 12864_2023_9955_MOESM9_ESM.png]

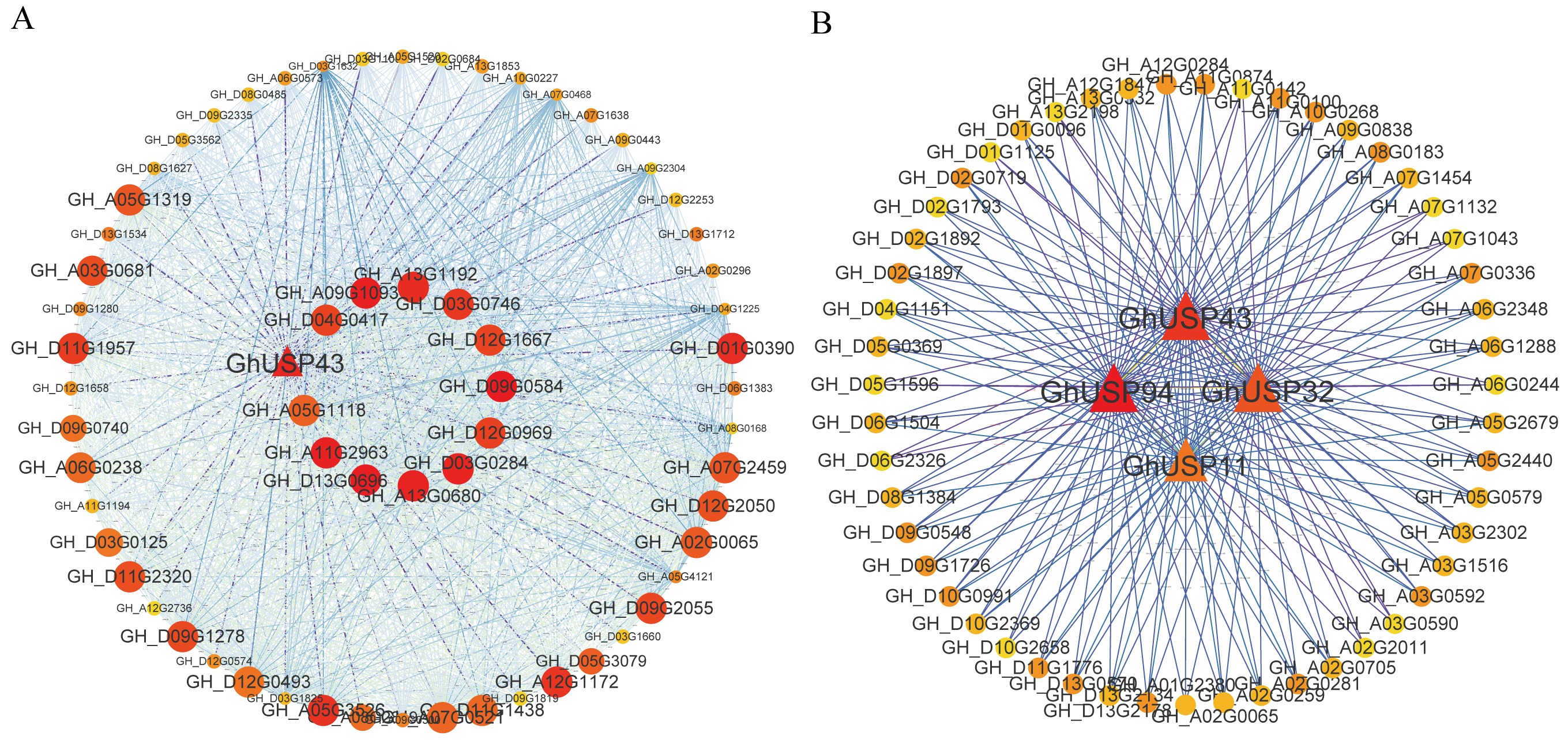

Supplement: Supplementary file 10 — Supplementary Material 10: Fig.S4. Gene co-expression network contained GhUSPs related to abiotic stress. (A) The co-expression network under salt treatment. (B) The co-expression network under PEG treatment. The red triangle symbolizes GhUSPs. The displayed genes and their corresponding description are presented in Table S4 and Table S5 [file 12864_2023_9955_MOESM10_ESM.png]
